# Exploring the Onset of a Male-Biased Interpretation of Masculine Generics Among French Speaking Kindergarten Children

**DOI:** 10.3389/fpsyg.2019.01225

**Published:** 2019-05-29

**Authors:** Pascal Mark Gygax, Lucie Schoenhals, Arik Lévy, Patrick Luethold, Ute Gabriel

**Affiliations:** ^1^Department of Psychology, University of Fribourg, Fribourg, Switzerland; ^2^Department of Psychology, Norwegian University of Science and Technology, Trondheim, Norway

**Keywords:** grammatical gender, gender representation, kindergarten learning, generic masculine, role noun

## Abstract

In French, and other gender marked languages, there are two ways to interpret a grammatical masculine form when used to refer to social roles or occupations [e.g., *les magiciens* (the magicians*_masculine_*)]. It can refer to a group composed of only men (specific use of the masculine form), or one composed of both women and men (generic use). Studies of adults revealed that the rule that masculine forms can be interpreted as inclusive of either gender is not readily applied. To gain a better understanding of the processes shaping this phenomenon, we present a follow-up study (*N* = 52) to Lévy et al. (2016) to explore how French-speaking kindergarten children (3–5 years of age) resolve the semantic ambiguity of the grammatical masculine form when presented with role or occupation nouns. In a paradigm where participants’ gazes were monitored, children were presented with pictures of a pair of two boys and a pair of one girl and one boy and were prompted to *Look at the [role noun_masculine__plural__form_]*. First, the results suggest a stereotype effect in that children more strongly directed their gaze toward the boy-boy picture for stereotypical male role nouns, but toward the girl-boy picture for stereotypical female role nouns. Second, in the non-stereotypical/neutral condition we did not find an indication of any own-sex preference (as in Lévy et al., 2016), but of an influence of the role nouns’ grammatical gender, in that children more strongly directed their gaze toward boy-boy pictures than toward girl-boy pictures. We suggest that a specific interpretation of masculine forms might already start to emerge between 3 and 5 years of age, while gender stereotypes are still activated.

## Introduction

Research on the representation of gender in language has mainly focused on adults, yet focusing on children could document the onset of the intricate interaction between information provided by language structure, and by information transmitted through cultural processes in forming gender representations.

In the present study, we therefore examine the role that grammatical gender plays when French-speaking kindergarten children process role nouns in the masculine form. French, as with other grammatical gender languages (e.g., German and Italian), is a language that grammatically marks the sex of the referent in role nouns (e.g., *éducatrice/éducateur* – female/male educator). In cases where the masculine form is used, it can be interpreted either as a generic [i.e., when the sex of referent(s) is either unknown, irrelevant or when either sex is present] or specific [i.e., when the sex of the referent(s) is male]. Research on adults (e.g., Gygax et al., 2012) and adolescents (Chatard et al., 2005; Vervecken et al., 2015) has revealed, however, that representations of role nouns in the masculine plural form are frequently male-biased, which has been interpreted as indicating a gender specific interpretation of the form. It remains unclear at what stage of language acquisition this bias unfolds, as even though some research has been dedicated to French-speaking children’s early understanding of grammatical gender in general (e.g., Van Heugten and Shi, 2009; Royle and Valois, 2010; Cyr and Shi, 2013), little research has been specifically dedicated to the grammatical gender of role nouns (see Lévy et al., 2016, for an overview).

Research on grammar acquisition in French suggests that abstract grammatical gender categorization is acquired at an early stage. Cyr and Shi (2013), for example, showed that infants have abstract knowledge of determiner of gender classes by 20 months of age, and that the knowledge of gender feature and agreement (i.e., knowing what makes a grammatically feminine determiner or noun and perceiving when they match mismatch) is already robust by 30 months of age. However, whereas the assignment of gender for French nouns in general is largely arbitrary – hence cannot be acquired through semantics – the situation is different for role nouns, as their assigned grammatical gender signals the sex of the referent(s) [e.g., *une musicienne*_feminineform_ (a female musician); *un musicien*_masculineform_ (a male musician)]. The masculine form, however, can also be used for unknown referent(s), or to refer to a group composed of both women and men. Furthermore, a role noun’s grammatical gender can be signaled by its determiner [une_feminineform_ artiste vs. un_masculineform_ artiste (a female and male artist)] and/or by suffixes (e.g., *– euse*_feminine_*, – atrice*_feminine_*, – ière*_feminine_*, – eresse*_feminine_ vs. *– eur*_masculine_*, – ateur*_masculine_*, – ier*_masculine_). The grammaticalization of referent sex has been criticized for contributing to a binary view of gender and the asymmetric use of feminine and masculine forms as contributing to the reduction of women’s visibility (Gabriel et al., 2018). We argue that these linguistic devices and their related issues might impact children’s social development – at least in terms of social identity (Gabriel and Gygax, 2016) – compelling us to better understand when grammatically masculine forms start to bias gender representations of role nouns.

Exploring the onset of this bias, Lévy et al. (2016) used a preferential looking paradigm on 24- and 36-month-old children. In a preferential looking paradigm, children are typically presented with a set of stimuli, and their gazes are monitored to examine which stimulus or stimuli they tend to prefer to look at (depending on the task at hand). In Lévy et al. (2016), while being auditorily prompted with *Look at the [a role noun]*, children were presented with two pictures, each depicting two figures with attributes of the given role noun (e.g., mechanics with *blue overalls*). All role nouns were spoken in the masculine plural form and varied in whether they were female stereotyped (e.g., nurses), male stereotyped (e.g., mechanics), or non-gender stereotyped/neutral (e.g., musicians). In accordance with a specific interpretation of the role nouns, one picture presented two boys (*boy-boy*), whereas the other, in accordance with a generic interpretation of the role noun, presented a girl and a boy (*girl-boy*). The literature suggests that children at that age are already sensitive to gender stereotypes (e.g., Kuhn et al., 1978) and the children’s gazes in the male and female stereotyped condition were hence used to identify their preference for novel vs. familiar information (i.e., some children prefer looking at novel stimuli whilst others prefer looking at familiar ones), an issue that is particularly relevant for the age group under investigation. Analyzing children’s gazes in the non-stereotypical/neutral condition revealed an own-sex preference, but no indication of an influence of the role nouns’ grammatical gender. In sum, children were biased toward stereotypical representations of gender, and when no stereotype was present, they were biased toward their own sex.

Although gender stereotyping has been of interest since the 1960s, only recently have researchers shown precocious *understanding* of gender labeling. In this line of thinking, and although Lévy et al. (2016) showed only little impact of the masculine grammatical form on gender representation in 2- to 3-year olds (i.e., the children were either biased toward stereotypical representations of gender, or when no stereotype was present, they were biased toward their own sex), Lew-Williams and Fernald (2007) showed that 2- to 3-year-old Spanish-speaking toddlers could fully understand grammatical gender labeling. However, *understanding* grammatical gender and gender labeling may only be part of the factors explaining the effects of grammatical gender on mental representations of gender. Production, or more precisely spontaneous production, may also be important when considering representational biases. For example, considering the morpho-phonological cues in French, gender marks have been shown to be correctly inflected in production (i.e., not just comprehension) by French-speaking 4-year-old children (e.g., Karmiloff-Smith, 1979), whereas it has been suggested that gender-marked articles were correctly attributed to non-words’ endings (non-words with feminine or masculine suffixes such as *surbelle_femininesuffixes_* or *rinloir_masculinesuffixe_*) as early as 3 years in French (Karmiloff-Smith, 1979; Marchal et al., 2007). The same ages have been reported in other languages, such as Spanish (e.g., Pérez-Pereira, 1991) and German (e.g., Mills, 1986). Studies in children with language impairment have also shown that typical Spanish-speaking 3-year-old children (Bedore and Leonard, 2001) and typical Swedish-speaking 2½-year-old children (Leonard et al., 2001) correctly inflect spoken *adjective-noun* and *article-adjective-noun* forms.

In the present study, we aim to conceptually replicate and extend the study by Lévy et al. (2016) by expanding the age-range to kindergarten children (age 36–70 months). More specifically, we investigate whether the masculine form will be considered as a mark referring to men, mimicking the male bias found in adolescent and adult populations. We expect that the masculine form will be considered as a mark referring to men, mimicking the male bias found in adult populations. In terms of the paradigm used, similar to that of Lévy et al., 2016 we essentially expect our participants’ gazes to be drawn toward the picture representing two boys (as opposed to that representing a girl and a boy), when orally prompted by the sentence “*Look at the [role noun*_masculine plural form_].*”*

## Methods

### Participants

**Figure 1:**
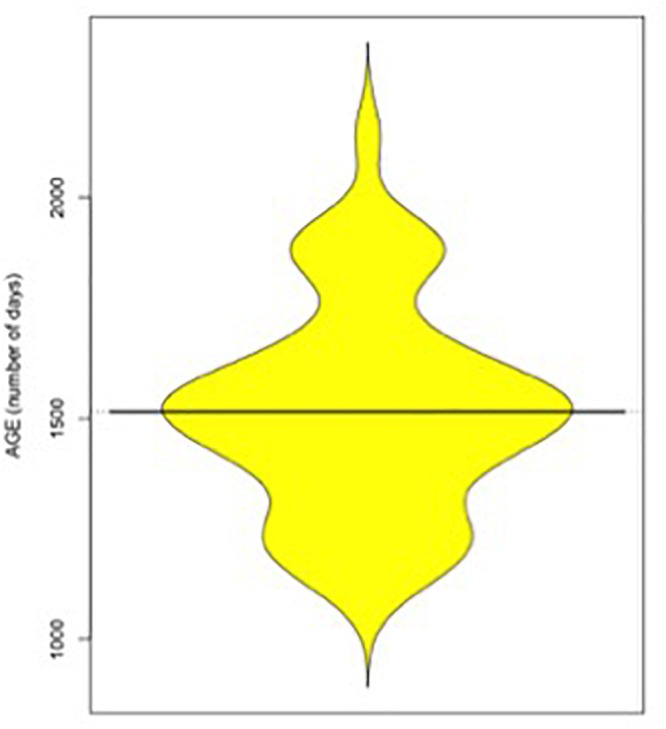
Beanplot of the age distribution.

Participants were 52 French-speaking children (27 girls and 25 boys) aged between 3 years and 6 days to 5 years and 314 days (*M* = 4 years and 55 days; *SD* = 234 days). Although we were looking for different ages, as in Lévy et al. (2016), the distribution of age was centered around the age of 4 (i.e., 1500 days). We could therefore not separate our sample into two clear age groups (see Figure 1 for a *beanplot* of age distribution). All children were recruited from nurseries and kindergartens in Lausanne and Geneva (Switzerland). Two boys had to be removed from the analyses, as, due to a technical issue, post-prompt data were not recorded. This study was approved by the Ethics Committee of the Department of Psychology (University of Fribourg) and carried out in accordance with their recommendations. All participants’ parents granted written informed consent for their child to participate.

### Materials and Procedure

#### The Setting

##### General setting

The experiment was always conducted in the kindergarten that the children attended. All locations were set up as similarly, as possible, and children were tested in a quiet place.

##### The eye-tracker setting

As in Lévy et al. (2016), the setting was composed of a chair for the referent, placed 40 centimeters in front of a 15-inch presentation screen and a loudspeaker placed on a table. The computer for controlling the time course of the item presentations was placed behind the referent, so that the child was not distracted. Eye movements were recorded using a desktop-mounted, video-based infrared eye-tracking system (Eyelink 1000, SR Research Ltd, Ontario, Canada) with a spatial resolution of 0.1° and a temporal resolution of 500 Hz. The experiment was written and controlled with the Experiment Builder Software^©^ (Eyelink I, SR Research Ltd, Ontario, Canada).

#### The Experiment

Participants were presented with 30 role nouns in the (plural) grammatical masculine form (see Table 1 for the full list of role nouns used), which were each accompanied by a picture and an auditory prompt. In Lévy et al. (2016) some role nouns, normed for their stereotypicality by Gabriel et al. (2008), were very poorly understood, at least according to parents’ reports. We removed those and if possible replaced them with similarly, stereotyped ones [as indicated by the more recent norming study by Misersky et al., 2014; e.g., *conducteurs* (drivers) was replaced with *conducteurs de taxi* [(taxi drivers)]. The resulting 30 role nouns were female stereotyped (e.g., *nurses, cashiers*, and *hairdressers*), male stereotyped (e.g., *boxers, hunters*, and *taxi drivers*), or non-stereotyped (e.g., *pedestrians, musicians*, and *neighbors*). In French, most role nouns [with the exception of auteurs_masculine_ – autrices_feminine_ (male and female authors)] in this study follow the principle by which the feminine form is a combination of the masculine form and an added or changed suffix. Note that we did not present any role nouns in the feminine form, as the meaning of the feminine form is unambiguous, and would require us to have an avatar composed of two girls. In terms of the rationale of our study – how the ambiguity of the masculine form is resolved – such a combination would not have been pertinent.

**Table 1:** The list of French role nouns used in the experiment.

French	English translation	Stereotype norms (SD)
**Female stereotypes**		
Babysitters	*Babysitters*	0.90 (*0.09*)
Caissiers	*Cashiers*	0.75 (*0.13*)
Coiffeurs	*Hairdressers*	0.75 (*0.11*)
Danseurs	*Dancers*	0.68 (*0.11*)
Danseurs de ballet	*Ballet dancers*	0.76 (*0.17*)
Infirmiers	*Nurses*	0.72 (*0.13*)
Maîtres	*Teachers (Kindergarten)*	0.80 (*0.13*)
Mannequins	*Fashion models*	0.68 (*0.15*)
Nettoyeurs^∗^	*Cleaners*	0.43 (*0.26*)
Patineurs artistique	*Figure skaters*	0.65 (*0.13*)
**Male stereotypes**		
Boxeurs	*Boxers*	0.20 (*0.16*)
Chasseurs	*Hunters*	0.15 (*0.11*)
Conducteurs de taxi	*Taxi drivers*	0.18 (*0.14*)
Constructeurs^∗∗^	*Builders*	0.10 (*0.09*)
Fermiers	*Farmers*	0.27 (*0.13*)
Footballeurs	*Footballers*	0.21 (*0.12*)
Magiciens	*Magicians*	0.29 (*0.13*)
Mécaniciens	*Mechanics*	0.20 (*0.16*)
Pêcheurs	*Anglers*	0.18 (*0.11*)
Prisonniers	*Prisoners*	0.31 (*0.14*)
**Neutral stereotypes**		
Auteurs	*Writers*	0.46 (*0.1*)
Boulangers	*Bakers*	0.41 (*0.17*)
Coureurs	*Runners*	0.45 (*0.12*)
Écoliers	*Schoolchildren*	0.53 (*0.07*)
Joueurs de tennis	*Tennis players*	0.44 (*0.09*)
Musiciens	*Musicians*	0.47 (*0.08*)
Nageurs	*Swimmers*	0.44 (*0.1*)
Piétons	*Pedestrians*	0.52 (*0.04*)
Skieurs	*Skiers*	0.43 (*0.1*)
Voisins	*Neighbors*	0.51 (*0.05*)

*Stereotype norms, in the form of the proportions of women perceived in the occupations, come from Misersky et al. (2014), except when stated otherwise. ^∗^Initially, as the study was also aimed at a comparison with English, we compared the French norms to English ones, which showed a mean of 0.85 (0.15). We therefore decided to keep the role noun in the male stereotype group. ^∗∗^This role noun was not present in Misersky et al. (2014) but was evaluated in Lévy et al.’s (2016) Pilot study as male [i.e., 0.35 (0.12)]*.

**Figure 2:**
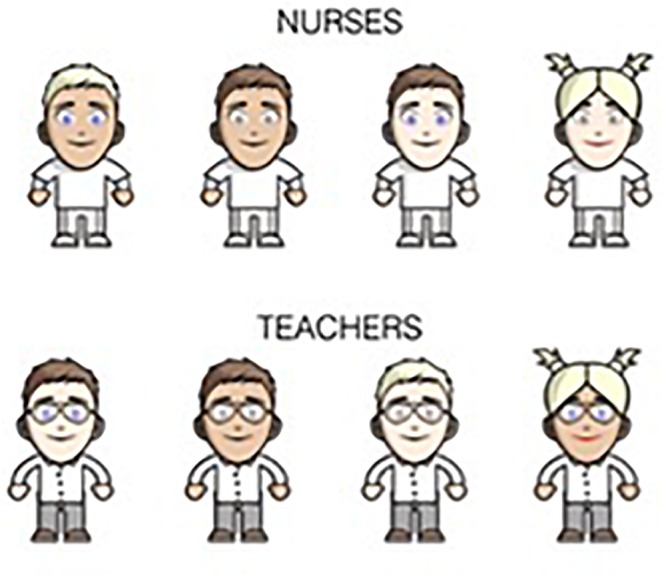
Examples of the avatars used in the experiment.

The pictures were therefore each composed of four characters divided into two pairs, which always consisted of a pair of two boys (referred to as boy-boy) and a pair of one girl and one boy (referred to as girl-boy). Most characters were taken from Lévy et al. (2016), while the remaining were created for the present experiment. Avatars were created with the www.doppelme.com toolbox and modified with Gimp 2.6.11. The characters held attributes of a given role noun (e.g., a sports shirt for *tennis players*, a guitar for *musicians*) that were not trivial at first glance but became obvious once the role noun was voiced (i.e., as confirmed by a panel of five judges from Lévy et al., 2016). This was important, inasmuch as we did not want participants to activate role nouns and/or show preferential gazes before the auditory prompt. Eye color (blue or brown), hair color (black or brown), and skin color (light or dark) were randomly assigned to each avatar. In essence, all avatars for a given role noun were similar but not identical, yet their main differences were whether they portrayed girls or boys (stereotypical eyes, mouth, and hair style attributes) and the nature of their occupational attributes (see Figure 2). In a pilot study (*N* = 8), children were shown all avatars – by role noun (i.e., 4 avatars per role noun) – and were asked to point at *the girls or the girl* within the 4 avatars. All eight children correctly and easily identified only one girl per role noun.

In the experiment, each item was presented to the participants in the following order: (1) a girl-boy and a boy-boy picture, and (2) an auditory prompt whilst the pictures remained on the screen. Before the auditory prompt, the boy-boy and girl-boy pictures were presented simultaneously, one on the left and one on the right (the position was randomly assigned for each role noun and each participant). The duration of the pre-prompt phase was gaze contingent, in that participants had to look at both sides of the screen (i.e., both pictures) for *at least* 2 s. Note that if a child’s gaze was drawn to only one of the pictures (i.e., the child would look at one picture for more than 2 s, whereas they would not look at the other one), the experimenter would remind them to look at both pictures. As soon as both pictures had been looked at for at least 2 s each, the auditory prompt would tell them to *Look at the [role noun_pluralform_]* [in French: *Regarde les_grammaticallyneutral_* (*role noun_masulinepluralform_*)]. So, for example, the prompt would say *Regarde les musiciens_masculinepluralforms_* [Look at the musicians], whilst showing two pictures, one with a girl and a boy, and one with two boys. Prompts had been recorded (44.1 kHz, 16 bits, stereo) either with a male voice, or with a female voice with Sound Studio 3 in an IAC booth. As length slightly varied from item to item, we used Audacity to adapt all items so that they would last 1.8 s and sound similar. Participants were randomly assigned to hear a male or female voice (58% of boys and 48% of girls heard a female voice).

To catch participants’ attention and center their gazes, each item was preceded by three colored lights flashing in the center of the screen with a bell ringing. Once a participant’s gaze was centered, the experimenter manually initiated the presentation of a stimulus on the monitor.

As the prompt was generated, the pictures stayed on the screen for 4 s. Gaze fixations were recorded during both pre-prompt and post-prompt phases.

#### Awareness of Gender Derivational Suffixes

In French, role nouns are declined in accordance to referents’ gender and contain feminine suffixes when a group is composed exclusively of women (e.g., *-euses, -onnes, -ères*, and *-ennes* etc.), and contain masculine suffixes (e.g., *-eurs, -ons, -ers*, and *-ens*, etc.) when the group is composed either of only men or men *and* women. Importantly, in Lévy et al. (2016), the lack of grammatical gender effects may be imputed to the rather poor knowledge children may have had in gender derivational suffixes. Therefore, to check children’s explicit awareness of gender derivational suffixes, we created two computer-assisted tasks: a comprehension and a production derivational suffix task. Both tasks were administered after the experiment. Due to time constraints, one child completed only the derivational suffix comprehension task and eight children completed neither task. The children’s performance in the production task was consistently poor, such that we did not further explore it.

In the *derivational suffix comprehension* task, children were presented with two boxes, one labeled with a female avatar and the other with a male avatar. The experimenter explained that the children would view pictures of fish, would be told the name of each fish and it would be their task to decide whether each fish was male or female and to place them in the appropriate box (i.e., female vs. male). A higher awareness of gender derivational suffix would result in a higher number of fish being categorized according to the gender derivational suffix of the fish’s name.

**Figure 3:**
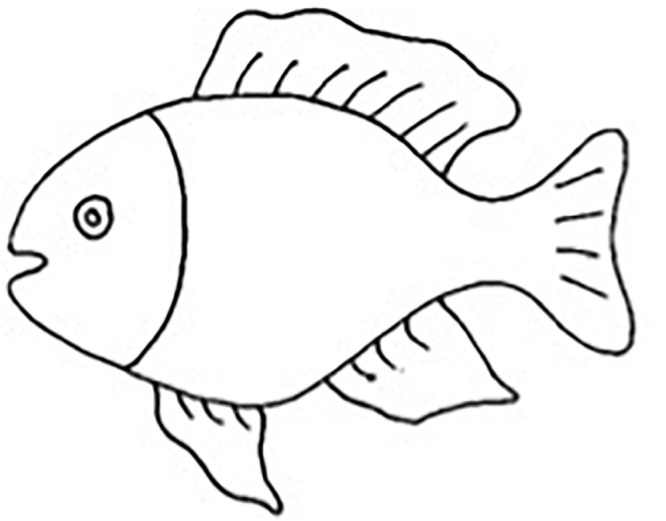
An example of a gender-neutral fish used in the Derivational Suffix comprehension task.

As shown in Figure 3, the pictures of fish were black and white drawings and were chosen by five adult judges to be as gender-neutral as possible. The names of the fish were created by adding a gender marked derivational suffix (out of five possible derivational suffixes) to a mono- or a bi-syllabic pseudo-word (e.g., doubier*_masculine_* – doubière*_feminine_*, rouffiteur*_masculine_* – rouffitrice*_feminine_*). This procedure is often used in tasks that evaluate verbal short-term memory with a non-word repetition task (Majerus, 2012).

In the actual task, three habituation items were followed by ten test items. The habituation items consisted of a fish for which familiar names were used: *mummy* or *daddy*, *mister* or *madam*, as well as the participant’s first name. The experimenter would make sure these items were correctly classified before moving on to the test phase. The test items consisted of ten fish-pictures, five of which were combined with names with a male derivational suffix, and five of which were combined with names with a female derivational suffix. The ten items were presented in a random order per participant, and the children’s responses were hand coded as *correct/incorrect* during the task by the experimenter.

The task showed that, on average, although our participants’ scores were not optimal, they did slightly better than guessing randomly [*M* = 6.12; *SD* = 1.61, 95% CI (5.62, 6.62)]; range: 3–10). Performance was not related to age [*r* = 0.14, *p* = 0.37, 95% CI (-0.17, 0.43)] and there was no significant sex difference [*M*_girls_ = 6.41, *M*_boys_ = 5.80, *t*(40) = 1.23, *p* = 0.23].

## Results

### Proportion of Gazes on the Boy-Boy Picture

Complying with the original method (Fantz, 1958, cited by Houston-Price and Nakai, 2004) and in line with Lévy et al. (2016), total fixation times per picture were used for our analyses. As in Lévy et al. (2016), the *proportion of gazes on the boy-boy picture*, computed as the gaze time spent on the *boy-boy* picture relative to the total amount of gazes spent on both pairs (the *girl-boy* and the *boy-boy*), were included in the analyses as the proportions are complementary (i.e., a proportion of 0.3 on the *boy-boy* meant a proportion of 0.7 on the *girl-boy*).

**Figure 4:**
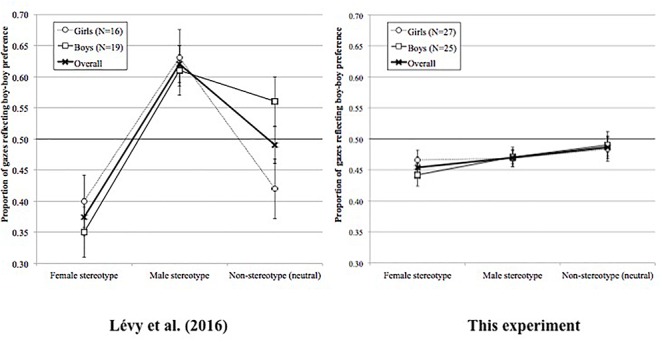
Proportions of gazes in Lévy et al. (2016); ^©^ Reprinted with permission of Cambridge University Press) and in the present experiment reflecting a *boy-boy* preference in all three stereotypical conditions overall and split by sex of respondent.

A general 3 (Stereotype: Female vs. Male vs. Neutral) × 2 (Sex of participant: Girls vs. Boys) full factorial ANOVA with Sex of participant as a between-subject factor and Stereotype as a within-subject factor was run on the proportion of gaze time on the *boy-boy* pictures. The analysis revealed no main effect of Sex of participant (*F* < 1), no main effect of Stereotype, *F*(2,96) = 1.64; *p* = 0.20, and no interaction effect (*F* < 1). Interestingly though, contrary to Lévy et al., 2016 the proportion of gazes to the boy-boy picture was always lower than 0.5, as shown in Figure 4, and the proportion of gazes to the girl-boy picture was always higher than that to the boy-boy picture.

We believe that there are three possible interpretations for these results. First, it could be that some children looked at those pictures that resemble things that they have often been exposed to in their daily life (familiarity effect), yet others might have looked at what appeared new to them (novelty effect). Essentially, these effects would cancel each other out ^1^. We carefully scrutinized the data to look for possible patterns signaling differences in gaze patterns illustrating *novelty* vs. *familiarity* effects (see Lévy et al., 2016 for a discussion on this issue). In the present data, there were no such signals. Importantly, in the neutral condition, irrelevant of the gaze pattern (i.e., familiarity or novelty), the results should have been at 0.5 (i.e., no stereotype effect), which was not the case. Second, when prompted with a role noun in the masculine form, our participants may have dominantly considered it as a generic form, which would indicate a different pattern than that in younger children (e.g., Lévy et al., 2016), but also different than that in adolescents (e.g., Vervecken et al., 2015), and adults (e.g., Gygax et al., 2012). However, before embracing the latter interpretation, a third one needs to be considered, that is the possibility that the girl-boy picture simply took longer to process, as there were two different genders represented, and that such a process blurred the results.

To check this third interpretation, we analyzed each participant’s pre-prompt gaze behaviors by narrowing the area of interest to only include gazes toward the avatars. This analysis could not be performed by Lévy et al. (2016) due to the way eye movements were monitored.

### Pre-prompt Gaze Behaviors

#### Pre-prompt Gaze Proportions

**Figure 5:**
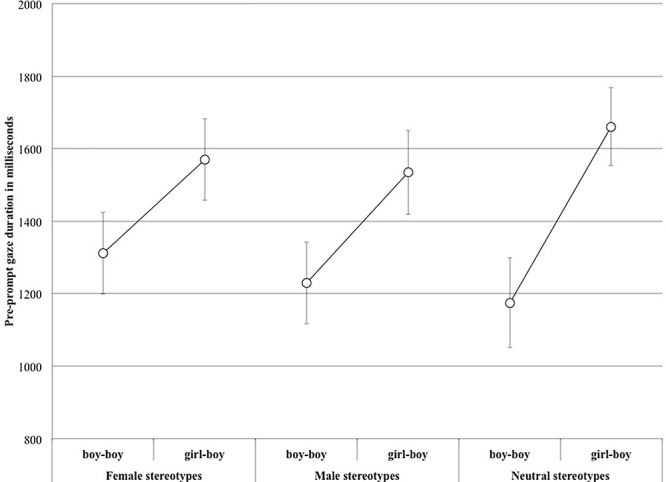
Mean pre-prompt gaze duration (and standard errors) across Picture and Stereotype.

The analysis of participants’ pre-prompt behaviors revealed that in the pre-prompt phase (i.e., without any prompt as to where to look) the girl-boy picture was looked at for longer than the boy-boy one (*M_boy-boy_* = 1239 ms, *SD* = 828; *M_girl-boy_* = 1588 ms, *SD* = 792; *t*(2994) = 11.80, *p* < 0.001; see Figure 5), indicating that the girl-boy picture did indeed take longer to process. Such a result compelled us to reconsider the analysis presented earlier and to use a somewhat different analysis strategy. Since we had a complete set of data, and, contrary to Lévy et al. (2016), we did not have to hand-code some of our data, we decided to depart from gaze proportions.

#### Pre-prompt Gaze Times

**Figure 6:**
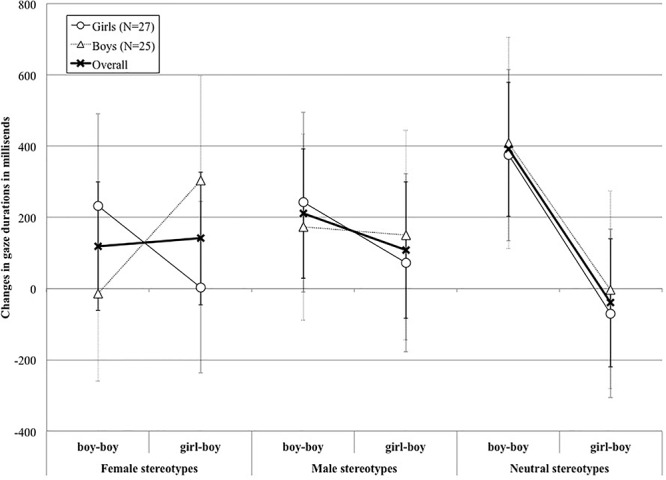
Mean *pre-post prompt* changes (and standard errors) in gaze durations across conditions. Positive times mean more gazes at the post-prompt phase.

We computed per participant for each trial a differential time by subtracting the total fixation time per picture in the pre-prompt phase from the total fixation time per picture in the post-prompt phase. This subtraction accounted for the fact that, without prompt, the *girl-boy* picture took longer to process. A resulting positive time duration meant that participants increased their gaze in the post-prompt phase compared to the pre-prompt one (see Figure 6). All analyses were run on these differential times.

In order to account for pre-prompt particular patterns and to also include both participants and items as random factors in all analyses (Clark, 1973; Brysbaert, 2007), we analyzed the data by fitting linear mixed-effects models using the R software (R Development Core Team, 2010, version 3.1.2). Models were tested using the *lmer*() function of the lmer4 package of R, and model comparisons were assessed using the *anova*() function, which calculates the Chi-square value of the log-likelihood in order to evaluate the difference between models, following Baayen’s (2008) procedure. As done in other studies on similar issues (e.g., Öttl and Behne, 2016) models were compared using a forward-testing approach. Fixed effects were included one at a time, and each resulting model was compared to a model that did not include the added factor. When comparing models, we also evaluated the contribution of random slopes to the models by using log likelihood tests (if the random slopes were justified by the design, as recommended by Barr et al. (2013). In fact, for all tested models, the inclusion of random slopes was not warranted (i.e., did not improve the models). We therefore retained only participants and items as random intercepts for our random structure. Finally, to obtain *p*-values for our final model, we used the *summary*() function from the lmerTest package (Kuznetsova et al., 2014).

When comparing our random model – only encompassing items and participants as random factors – to one also including Picture (boy-boy vs. girl-boy), the latter showed a better fit, Δχ^2^ = 12.731, Δdf = 1, *p* < 0.001. This model was further improved by adding Stereotype (Neutral vs. Male vs. Female), Δχ^2^ = 16.744, Δdf = 4, *p* < 0.01, accounting for main and interaction effects. The model fit was not improved by adding Age (as a continuous variable), Sex of participant (Girls vs. Boys), Voice (Female vs. Male), or Derivational Suffix Comprehension.

We therefore kept the model that included Picture and Stereotype as fixed factors and their interactions, and participant and item as random intercepts as our final model. The estimates of this model are shown in Table 2, and the means for the *pre-post prompt* changes in gaze durations across conditions are shown in Figure 6. For the Stereotype factor, the contrasts for our model were calculated with the Neutral condition as the base level. Essentially, the Neutral condition was the only condition bearing only one potential effect, that of grammatical gender (and not stereotype). For our Picture factor, the contrasts for our model were calculated with the boy-boy picture as the base level.

**Table 2:** Model estimates for our best fitting model, including Picture and Stereotype as fixed factors (main and interaction effects) and participants and items as random intercepts.

	Estimate	*SE*	*df*	*t*-value	Pr(> | t|)
Intercept	391.27	60.39	784	6.479	<0.001
Picture					
Girl-boy	-430.96	82.41	2421.9	-5.229	<0.001
Stereotype					
Male	-180.57	82.45	2422	-2.19	<0.05
Female	-272.07	82.45	2422	-3.3	<0.001
Picture^∗^stereotype					
Girl-boy and male	328.86	116.61	2421.9	2.82	<0.01
Girl-boy and female	453.29	116.61	2421.9	3.887	<0.001

*Treatment contrasts were used for all unordered factors (i.e., Stereotype). The Neutral condition is set as the baseline level*.

The estimates of the model showed several effects. First, the gaze patterns to the girl-boy picture were different to that to the boy-boy one. More specifically, participants significantly increased the length of their gazes to the boy-boy picture (240 ms) after the prompt than they did to the girl-boy picture (70 ms). Second, the general increase in gaze duration was different across stereotype conditions. Namely, it was bigger in the neutral stereotype condition (176 ms) than in the male condition (159 ms) or the female condition (130 ms). Third and most importantly, the difference in gaze increase between the boy-boy and the girl-boy pictures differed between the neutral stereotype condition and the male one, as well as between the neutral stereotyped condition and the female one. As illustrated in Figure 6, the male bias effect in the neutral stereotype condition (boy-boy: 391 ms; girl-boy: -40 ms) – although being significantly stronger to that in the male stereotype condition (boy-boy: 211 ms; girl-boy: 108 ms) – was in the opposite direction to that of the female stereotype condition (boy-boy: 119 ms; girl-boy: 141 ms).

## Discussion

In their experiment in French, Lévy et al. (2016) showed that children aged 2–3 were mainly driven by stereotypes when processing role nouns presented in the masculine grammatical form. Their experiment contrasted with those in adults (e.g., Esaulova et al., 2014, in German; Garnham et al., 2012, in French and German; Gabriel and Mellenberger, 2004, in German; Gygax et al., 2008, in French and German; Stahlberg et al., 2007, in German) mostly showing that adults struggle to process the masculine form as a generic one, and tend to have a higher tendency to attribute men to role nouns or occupations written in the masculine form, in most cases regardless of stereotype.

In this paper, we hypothesized that 3–5 year old children, although still influenced by stereotypes, would gradually start to be influenced by the specific meaning of the masculine grammatical form. The results of the experiment supported our hypotheses. In all, the children were more likely to increase the length of their gaze toward the *boy-boy* picture after having been prompted by a role noun in the masculine form. This effect was particularly strong when role nouns carrying no stereotypes were shown. In other terms, when no stereotypes are associated with a role noun, children look for different cues to assign gender. More specifically, they rely on the grammatical form of the role noun, and in our case, a *specific* interpretation of the masculine grammatical form.

The general male bias in our data is striking, as it appears at a kindergarten period, meaning before children are formally instructed about grammatical gender. As our grammatical gender awareness scores did not show full awareness of grammatical gender markings, we can only assume that the masculine bias that became apparent in our data may only be fully active as soon as children start to completely master the idea that the masculine form is used to refer to men, and that the feminine form is used to refer to women (e.g., gender derivational suffixes). These rules may only be fully acquired when formally taught.

In light of the male biases found in the literature on both older children and adults (Children: Hyde, 1984; Chatard et al., 2005; Vervecken et al., 2015; Adults: Stahlberg et al., 2007; Garnham et al., 2012; Esaulova et al., 2014), we doubt that at any moment in the development of grammatical gender awareness will language users be able to fully activate (at least spontaneously) the generic interpretation of the masculine form (learnt later on), at least never at the expense of the specific meaning of it. Gygax et al. (2012) even suggested that the specific meaning of the masculine form was activated, at least for adults, in a passive way (i.e., without control), and that the generic meaning had to be consciously and strategically activated. Their suggestion was actually also based on the fact that children formally learn the specific meaning of the masculine form before the generic one (see Gygax et al., 2009, for a discussion on the formal learning of grammatical gender). Consequently, one could argue that children are more (i.e., more often and from earlier ages) exposed to the masculine form’s specific meaning. Our data provide further evidence that the specific meaning of the masculine form seems to be learned even informally, between 3 to 5 years of age, suggesting that children are more often spoken to using the specific meaning of the masculine form.

In terms of stereotypes, our data also show that stereotypical knowledge still plays a role, as in Lévy et al., 2016 in assigning gender. In fact, although participants seemed to have been influenced by the specific meaning of the masculine form when processing gender-neutral occupations, the effect was very different for female stereotyped ones, hinting at simultaneous effects of stereotypes and grammar for these role nouns. Note that we expected the effect on masculine stereotyped ones to be stronger, as both grammar and stereotype allegedly force participants’ representation in the same direction (i.e., male bias). We believe that this illustrates the variation in the way some of the male role nouns are perceived, in terms of stereotypes. For example, girls’ gazes seemed to be more attracted to the girl-boy picture when presented with the male stereotyped role noun *taxi drivers* [chauffeur de taxi], and boys’ gazes were drawn to the girl-boy picture when presented with the male stereotyped role noun *anglers* [pêcheurs]. Although we do not wish to enter into this debate in detail, it may be the case that some of the male stereotyped role nouns have changed, or are changing in society, leading our data to show stronger noise for them.

The systematic prominence of stereotypes for our participants is not surprising, especially in light of the results of Lévy et al. (2016), as well as those of others, who found a particular sensitivity to gender stereotypes at a very early age. For instance, in Poulin-Dubois et al. (1998), 24-month-old toddlers associated gender stereotypical toys (e.g., a *doll* or a *car*) with faces of girls and boys. Others (e.g., Eichstedt et al., 2002; Serbin et al., 2002), using the same paradigm as we did, showed that 24-month-old toddlers already looked longer at activities that were inconsistent than those that were consistent with gender norms (e.g., looking longer at the man putting on lipstick than at the woman).

In sum, and concretely, we have shown that parts of the mechanisms underlying the understanding of the masculine form are implicitly learnt (i.e., in the kindergarten period). As suggested by Zosuls et al. (2009), we believe that children’s exposition to semantic and morphophonological cues generates particular associations, and that the associations *masculine = men* are more frequent than *masculine = generic*. Of course, empirical evidence to support this frequency exposition is needed. Our data only represent an indirect signal of this possible imbalance. We can still suggest that children start to learn, understand and produce correct gender inflections, at least in French, between 3 and 5 years of age, and that this has an impact on the way they process role nouns presented in the grammatical masculine form. That is, when processing the masculine grammatical form in French, although interpretable as a generic form, children aged 3–5 years tend to start showing similar male biases as adults do.

As a final note, we would like to stress three issues important for future research on this topic. First, in our study, we tried to understand the mechanisms involved when children process the masculine form by prompting children’s gaze with role nouns in the masculine form. Roles nouns – nouns that do not carry gender as their core meaning, such as *queen* – are particularly well suited for the investigation of both *semantic* as well as *grammatical knowledge* development, and we would argue that their processing may not undergo the same development as nouns that do not explicitly refer to humans (e.g., inanimate beings). Second, future research could also focus on comparing different linguistic forms, such as pair-forms – using both the feminine and masculine form to refer to one referent (as in Vervecken et al., 2015) – to better understand the true impact of the masculine form on young children. Last but not least, Gabriel et al. (2017) suggested that stereotype activation may be more prominent when understanding speech (i.e., as in the present experiment) than when understanding text. As such, it could be the case that the male-bias associated with the use of the masculine form may be stronger when children learn to read. Future research could compare speech and text comprehension directly to understand the developmental patterns associated with the understanding of the masculine form.

## Ethics Statement

This study was approved by the Ethics Committee of the Department of Psychology (University of Fribourg) and carried out in accordance with their recommendations. All participants’ parents have granted informed consent to their child participation.

## Author Contributions

PG and UG got the grant for this project. PG wrote the first draft of the manuscript. LS, AL, and PL prepared parts of the design, tested the children, and participated in the designing phases as well as the writing phases.

## Conflict of Interest Statement

The authors declare that the research was conducted in the absence of any commercial or financial relationships that could be construed as a potential conflict of interest.

## Footnotes

1 Houston-Price and Nakai (2004) suggest that data on binary preferential gazes should always be carefully scrutinized when testing young children. *Familiarity* vs. *novelty* effects may depend on age development, early preferences being illustrated by novelty, whereas later preference by familiarity. Houston-Price and Nakai (2004) even suggest that these gaze strategies may vary within the same experimental session for a given child.
